# Chemosensory Function in Burning Mouth Syndrome a Comparative Cross-Sectional Study

**DOI:** 10.3390/nu13030722

**Published:** 2021-02-25

**Authors:** Pia López-Jornet, Yolanda Collado, Alfonso Zambudio, Eduardo Pons-Fuster, Candela Castillo Felipe, Asta Tvarijonaviciute

**Affiliations:** 1 Faculty of Medicine and Odontology, Biomedical Research Institute (IMIB-Arrixaca) Hospital Morales Meseguer, Clínica Odontológica, Marqués del los Vélez s/n, 30008 Murcia, Spain; 2 Faculty of Medicine and Odontology, Hospital Morales Meseguer, Clínica Odontológica, Marqués de los Vélez s/n, 30008 Murcia, Spain; yolanda.cmurcia@gmail.com (Y.C.); azambudioabadia@gmail.com (A.Z.); candela.casti@gmail.com (C.C.F.); 3 Departamento de Anatomía Humana y Psicobiología, Faculty of Medicine and Odontology, Biomedical Research Institute (IMIB-Arrixaca), University of Murcia Spain, 30100 Murcia, Spain; eduardo.p.f@um.es; 4 Interdisciplinary Laboratory of Clinical Analysis INTERLAB, International Campus Excellence “Campus Mare Nostrum”, University of Murcia Murcia, 30100 Espinardo, Spain; asta@um.es

**Keywords:** burning mouth syndrome, smell, taste, OHIP-14

## Abstract

Taste and smell are considered to be functions that contribute to the maintenance of good nutritional status. The present study evaluates taste and smell function in patients with burning mouth syndrome (BMS) versus a control group. A cross-sectional study was made of 36 consecutive patients with BMS and 56 healthy patients. Smell was assessed using the Sniffin’ Sticks test, while taste was evaluated with Taste Strips. Oral quality of life was assessed with the Oral Health Impact Profile-14 (OHIP-14), and the severity of dry mouth with the Thompson Xerostomia Inventory. The patients with BMS had a mean age of 60.4 ± 10.5 years, while the controls had a mean age of 61.3 ± 19 years. No significant differences in smell were recorded between the two groups. In contrast, significant differences in taste function were observed between the patients with BMS and the controls. In the patients with BMS, 44.4% suffered taste alterations compared with the 3.4% healthy controls. Further studies in such patients are needed to allow improved management of the chemosensory problems, mouth dryness, and oral health-related quality of life in BMS.

## 1. Introduction

Burning mouth syndrome (BMS) is a chronic condition characterized by pain and/or burning sensation in the oral cavity, in the presence of an oral mucosa of normal appearance and with no local or systemic disorders [1,2,3]. The prevalence of BMS ranges widely from 1–14.8%, depending on the diagnostic criteria used [1,2,3]. The syndrome affects mainly women, particularly after menopause [1,2,3,4,5]. While the etiopathogenesis of BMS is not fully clear, there is growing evidence defining it as a neuropathic pain disorder and, thus, a direct consequence of a lesion or disorder affecting the somatosensory system [4,5], but the gustatory system has also been implicated in this condition [2].

Taste alterations are common in patients with BMS and manifest as distorted taste sensation, dysgeusia, or phantom taste perception [6,7]. Taste and smell disorders receive little attention but can adversely affect food intake and the choice of foods, giving rise to malnutrition, immune disorders, and worsening of diseases [8,9].

Smell sensation, such as the smell of a rose or lemon, is mediated by the first cranial nerve (olfactory nerve), while somatosensory sensations, such as warm, cold, or irritant, are mediated by the ophthalmic and maxillary divisions of the fifth cranial nerve (trigeminal nerve). The smell receptors are located within the olfactory neuroepithelium, a region found in the cribiform plate, superior nasal septum, and the posterior segment of the superior concha. The free nerve endings of the fifth cranial nerve are distributed throughout the nasal respiratory epithelium, including the regions of the olfactory neuroepithelium [9,10,11].

Taste sensation, in turn, is mediated by four peripheral nerve pairs: taste in the anterior portion of the tongue is dependent upon the chorda tympani, a branch of the seventh cranial nerve (facial nerve), while the posterior portion of the tongue and the pharynx are dependent upon the ninth cranial nerve (glossopharyngeal nerve). The soft palate in turn is dependent upon the superficial petrosal nerve (a branch of the seventh cranial nerve), and taste sensation in the larynx/esophagus is dependent upon the tenth cranial nerve (vagus nerve). The central taste pathways appear to be more divergent than the central smell pathways. Taste sensation is based on 5 different core flavors: sweet, salty, acid, bitter, and umami [5,6,7,12]. Parageusia and phantogeusia are qualitative taste disorders that cause distorted or hallucinatory taste sensations in the presence, or absence, of external stimuli, respectively. Collectively, taste disorders are grouped under the general terminology of dysgeusia, a term that generally refers to any kind of impaired taste sensation [13,14].

The overall oral sensory experience is the interaction of smell, taste, irritation, texture, and temperature and is defined as a perception resulting from the stimulation of a combination of taste buds, the olfactory organs, and chemoesthetic receptors within the oral cavity [10]. Indeed, this sensory interaction is what allows us to identify the different foods. Any factor capable of affecting taste, smell, or tactile sensation will affect the flavor of food. In this regard, saliva plays a role in the aforementioned sensory experience, contributing to the acceptance of flavors [8,15,16,17]. In this regard, carbonic anhydrase VI (CA VI), a secretory isozyme of the a-CA gene family highly expressed in the salivary and mammary glands and secreted into saliva, has been shown by many papers to be involved in taste perception, particularly bitter taste [18,19,20].

Taste alterations are found in 11–69% of all patients with BMS [21]. Of these subjects, 67–88% report phantom taste perception and 59–67% describe dysgeusia [22]. Phantom taste perception is described as bitter, metallic, or both. Acid and bitter taste may be described as more intense, sweet as more weak, and salty taste as more intense or weak than before onset of the disease [21,22,23,24,25].

Studies on taste detection thresholds and taste intensity in patients with BMS have confirmed the clinical findings of objective taste alterations in these patients [21,22,23,24,25,26,27,28]. In BMS, the salty and bitter taste detection thresholds are reported to be increased [21]. Patients with BMS also experience difficulties in identifying and remembering the quality of flavors [22]. A study involving taste strips found patients with BMS to be significantly less sensitive than the controls to different taste solutions, such as saccharose, citric acid, sodium chloride, and quinine hydrochloride [28].

Taste is closely linked to smell sensation, and any imbalance in this relationship affects patient quality of life and feeding habits [24]. The present study was carried out to evaluate taste and smell chemosensory function in patients with burning mouth syndrome (BMS) versus a control group.

## 2. Materials and Methods

The present study was conducted in accordance with the ethical principles of the Declaration of Helsinki and Good Clinical Practice and was approved by the Ethics Committee of the University of Murcia (Murcia, Spain).

All subjects provided written informed consent before inclusion, and their participation was coded throughout the course of the study. The reporting of data followed the STROBE (Strengthening the Reporting of Observational studies in Epidemiology) statement. The patients with BMS were recruited on a consecutive basis.

Inclusion criteria: patients over 18 years of age, diagnosed with BMS in department Oral Medicine of the University of Murcia according to the criteria of the International Headache Society (IHS) [29], which defines the disorder as an intraoral burning or dysesthetic sensation, manifesting for more than two hours daily over more than three months, in the absence of clinically evident causal lesions.

Exclusion criteria: patients with oral symptoms that could be attributed to conditions other than BMS, such as oral lichen planus, lichenoid reactions, candidiasis, drugs or diseases conditions known to affect taste sensation, patients receiving chemotherapy, individuals with upper respiratory viral infections, chronic diseases, such as gastroesophageal reflux or diabetes, vitamin deficiencies, patients with a history of middle ear surgery, oral infections, third molar surgery, the presence of materials causing contact sensitivity reactions, chronic smoking, and pregnant women.

The healthy controls were recruited from the Dental School of the University of Murcia, consisting of patients with the same sociodemographic characteristics as the patients with BMS, and matched for age and gender.

The participants were instructed to avoid eating, drinking, and smoking for at least one hour before the appointment. A trained professional in Oral Medicine compiled the case history and the conduction of complementary examinations. The duration of the disorder was also recorded.

Pain was scored using a visual analog scale (VAS) from 1–10 (0 = no pain, 10 = extreme pain), which consists of a straight line with the endpoints defining extreme limits, such as no pain at all and pain as bad as it could be. The subject is asked to mark his pain level on the line between the two endpoints to indicate the level of pain they experienced.

### 2.1. The Sniffin’ Sticks Test Smell Examination

Smell sensation was measured using the Sniffin’ Sticks test (Burghart Messtechnik, Wedel, Germany). The subjects identification test (were instructed to smell each of the 12 odor sticks and choose one of the four suggested answers describing the smell sensation) [30]. The hood of each stick was removed for 3–4 s, and the stick was placed approximately 2 cm from both nostrils of the patient. The participants were allowed to smell the stick repeatedly if they were not sure about the smell sensation. A 30-s interval was observed between the successive sticks. The participants were asked to choose a describing answer even if they smelled nothing or were not sure about the right answer—in this case rejecting the most unlikely answers. The test result was provided by the score of correct answers, based on a standardized classification: anosmia (score: 0–5), hyposmia (score: 6–9), and normosmia (score: 10–12)

### 2.2. Taste Examination

Taste sensation was examined after providing the participants with a detailed explanation of the test involved. Measures of taste function were obtained using validated filter paper strips impregnated with solutions covering four basic or core taste qualities: sweet, acid, salty, and bitter (sweet: 0.4 g/mL of saccharose; acid: 0.3 g/mL of citric acid; salty: 0.25 g/mL of sodium chloride; bitter: 0.006 g/mL of quinine hydrochloride) [31]. The taste strips (length 8 cm, area of the tip 2 cm^2^) (Burghart Messtechnik, Wedel, Germany) were gently rubbed onto the anterior portion of the extended tongue. The different taste qualities were presented on a random basis, and the subjects were asked to identify the taste in each case. An interval of about 25 s was observed between the successive strips. The participants were allowed to rinse their mouth with water during the test. A dichotomic scale was used to score taste perception (0 = correct identification of the taste, 1 = unable to identify the taste). Those patients with alterations were questioned about the presence of qualitative taste disorders or strange taste sensations in the mouth. The frequency (constant, occasional) of these alterations was recorded.

### 2.3. Oral Health Impact Profile-14 (OHIP-14)

This questionnaire, in its short version, was applied. The instrument consists of 14 items that explore different aspects of oral function and quality of life. The patients were questioned about problems relating to speaking, taste perception, eating discomfort, and problems with dentures. The score ranged from 0–56, with higher scores corresponding to poorer oral quality of life [32].

### 2.4. Xerostomia Severity Test

The Xerostomia Inventory, validated by Thompson et al. in 1999, was used. The test assesses the situation of dry mouth and consists of 11 questions that explore the frequency of appearance of the defined symptoms during the last four weeks, scored from 0–5 (0 = never, 5 = always), with a maximum score of 55 points [33].

### 2.5. Statistical Analysis

Normal data distribution was assessed by the Kolmogorov–Smirnov test. The Student *t*-test for independent samples was used to compare continuous variables with a normal distribution in both the patient and control groups. A chi-square test was used to compare dichotomous variables for smell or taste alterations in patients with BMS and healthy controls. Statistical significance was considered for *p* < 0.05. The SPSS version 19.0 statistical package for MS Windows (SPSS Inc., Chicago, IL, USA) was used throughout.

## 3. Results

The study sample consisted of 36 patients with BMS (6 males and 30 females) with a mean age of 60.4 ± 10.5 years, and 56 controls (13 males and 43 females) with a mean age of 61.3 ± 19 years. There were no significant differences between the two groups in terms of either gender (*p* = 0.449) or age (*p* = 0.788) (Table 1).

With regard to smell sensation, none of the subjects in either group presented anosmia. Two patients in the BMS group presented hyposmia, versus a single patient in the control group, the difference being not significant (*p* = 0.407).

In relation to oral quality of life as assessed by the OHIP-14, the BMS group had significantly poorer quality of life than the control group (27.5 ± 7.90 in the BMS group versus 10.39 mean ± 2.54 sd (standard deviation) and in the control group; *p* < 0.001). The BMS group also presented significantly greater problems referred to dry mouth, as evidenced by the Xerostomia Inventory (18.89 ± 10.6 in the BMS group versus 11.27 ± 0.73 in the control group; *p* < 0.001).

A total of 44.4% of the patients with BMS presented taste alterations, versus a 3.4% of the controls, the difference being statistically significant (*p* < 0.001). No significant differences were recorded in the identification of sweet (*p* = 0.731) and salty taste (0.382) between the controls and the patients with BMS, though significant differences were noted in the identification of acid (*p* = 0.035) and quinine hydrochloride taste (*p* = 0.047).

In the present study, the most frequent tastes in the BMS group were a metallic flavor at 11 (64%), followed by bitter sensation at 4 (23.5%), and others at 2 (11.8%). The taste was described as being present most of the day on a constant basis in 58.8% of the cases, and occasionally in 41.1%.

The duration of BMS was 2.24 ± 2.2 years, and the VAS burning sensation-pain score was 7.94 ± 1.89. Neither this score nor the duration of BMS affected identification of any of the flavors evaluated (Table 2).

## 4. Discussion

Few studies to date have analyzed taste function in patients with BMS, and publications on smell sensation are practically no existent. Smell is one of the most important evolutive defense mechanisms in humans. In the present study, we recorded no statistically significant differences in smell function between the BMS group and the controls. The intact olfactory system allows us to identify food in poor condition and to detect certain toxic gases in the environment, for example, thereby contributing to avoid health risks. Smell moreover plays a role in the experience of eating, emotions, and quality of life [9].

Reduced or distorted taste sensation is one of the characteristics of BMS. Our findings are consistent with those of other studies [21,22,23,24,25,26,27,28] in that the patients with BMS showed statistically significant differences in taste identification with respect to the control group (*p* < 0.001). The taste alterations most frequently indicated by the patients corresponded to metallic taste, followed by bitter taste.

An association between taste alterations and pain in BMS has been suggested. In some cases, these taste-pain interactions may be a consequence of damage to the taste perception system involving either the chorda tympani, with release from inhibition of the glossopharyngeal nerve (alterations in taste, tact, and pain), or to the trigeminal nerve (alterations in tact and pain) [25,34,35,36].

On the other hand, patients with BMS who have a greater density of fungiform papillae [36] compared to healthy controls are known as “supertasters” [37]. Although some studies have suggested that taster status may influence a different papillae density, others [38] have argued against the use of papillae density in predicting taste sensitivity and caution against imprecise use of the term supertaster

It has been shown that the intensity of pain characterizing BMS is correlated to the density of the fungiform papillae at the tip of the tongue, and those individuals with a greater number of taste buds may also present higher density pain innervation, with this possibly increasing the risk of suffering taste disturbances and oral pain. Other studies have reported no differences in the density of the fungiform papillae between patients with BMS and controls [23,34]. Thus, supertaster status could be attributable to differences in taste sensitivity, which varies among individuals.

On the other hand, it must be taken into account that some cases of alterations in taste sensitivity may be attributable to genetically inherited insensitivity to certain compounds such as phenylthiocarbamide (PTC). Sensitivity to PTC and to other thiourea compounds, such as 6-n-propylthiouracyl, is conditioned by genetic variations (e.g., single nucleotide polymorphisms (SNPs)) of the TAS2R38 gene of the taste receptor [39]. In this study, we use quinine hydrochloride as a proxy for bitter taste perception. However, quinine is known to activate only some (i.e., 9) of the 25 TAS2Rs and is not able, per se, to summarize the overall bitter taste perception [40,41]. Other studies [42,43]. have indeed used similar taste strips containing other bitter-tasting compounds, such as PTC/) and salicin [44], to investigate specific bitter perceptions

Studies on taste dysfunction in patients with BMS pose problems, particularly in relation to the diagnosis and classification of cases as corresponding to either primary or secondary BMS [1,2,3]. Some studies offer little or no information on patient selection or details of the methods used. There are few psychophysical studies on taste detection thresholds, though the existing publications consistently show deficiencies in taste perception and in the taste intensity scale among patients with BMS. No studies have compared different methods in terms of their clinical usefulness, repeatability, or reliability.

Braud et al. [45] contrasted taste perception in 21 patients with BMS versus 21 controls, based on electrogustometry thresholds, evaluating the function of the fungiform and foliate papillae of the tongue. The patients with BMS were seen to have alterations in fungiform and foliate papillae taste sensitivity.

Imura et al. [27], in turn, studied a group of patients with BMS and recorded a decrease in salivary flow and a higher acid taste threshold in these patients, thus offering a possible explanation for their frequent complaints of simultaneous xerostomia and dysgeusia.

In a recent study published in 2020, Su et al. [46] retrospectively analyzed taste function in BMS patients after treatment with clonazepam 0.25 to 0.5 mg/day, to determine whether pain reduction is associated to a change in taste function of these individuals. In this regard, the treatment of patients with BMS significantly lessened the pain symptoms, resulting in improved taste function.

Salivary flow is important for dissolving flavors within the oral cavity, and it also participates in maintenance of the taste buds [15,16,17]. The participation of saliva in taste sensation and perception is currently considered to be crucial. Although the components of food are largely responsible for these sensory characteristics, the composition of saliva can alter food perception. Mosca and Chen [16] examined how the interactions between saliva and food can influence different aspects of the oral perception of food, including taste and texture. Saliva participates in the oral sensory perception of food at different levels. It not only interacts with food but also constitutes a medium that bathes the oral structures. In recent years, there has been growing evidence that saliva makes an important contribution to the perception of both taste (flavor and aroma) and texture [16,17]. Specific salivary proteins have been identified and related to individual sensitivity to the basic or core flavors [47,48]. Walliczek-Dworschak et al. [48] observed a correlation between taste perception and the salivary flow rate. In this regard, alterations in salivary flow can modify the concentration of electrolytes, showing that such changes influence taste perception. In the present study, dry mouth was only assessed subjectively based on the Thompson Xerostomia Inventory, although significant differences were observed between the BMS group and the controls.

In 2020, Su et al. [49] evaluated the correlation between of geographic tongue upon taste, salivary flow and the pain characteristics of patients with BMS in order to determine whether geographic tongue contributes to BMS. The authors reported differences in age, gender, oral pH, smell, and pain between the groups. Stimulated and unstimulated salivary flow were both significantly lower in patients with BMS and geographic tongue. The responses to all the taste stimuli and to ethanol were significantly lower in patients with BMS, with the exception of bitter taste in the fungiform papillae. The investigators suggested that geographic tongue may be a factor in the development of BMS through a mechanism not implicating taste sensation.

Pereira et al. [50], in a systematic review and meta-analysis, show the influence of BMS upon patient quality of life. Loss of the pleasure of eating caused by BMS had a negative impact upon quality of life, in concordance with our own observations. In patients with BMS, pain not only has a strong sensory impact but is also characterized by an affective/motivational effect.

With regard to the limitations of our study, mention must be made of its cross-sectional design, which can give rise to bias. On the other hand, we explored taste sensation using standardized strips instead of electrogustometry, which offers a more precise evaluation of taste sensitivity. Another limitation of this study is the lack of assessments of dietary intake and body composition of the participants.

## 5. Conclusions

In the present study, we recorded no differences in smell function between the BMS group and the controls. The patients with BMS suffered taste alterations compared with the healthy controls. Further studies in such patients are needed to allow improved management of the chemosensory problems in BMS food preferences, dietary intake, and nutritional status and to design new strategies and interventions.

## Figures and Tables

**Table 1:** Description and comparison of demographic variables and habits of the two study.

	Total	Group		*p*-Value
		Control	Case	
**Gender**, *n (*%*)*				0.449
1 Male	19 (20.7)	13 (23.2)	6 (16.7)	
2 Female		43 (76.8)	30 (83.3)	
**Age**, *mean (SD)*	60.9 (16.3)	61.3 (19.1)	60.4 (10.5)	0.788
**Smoking**, *n (%)*				0.933
Non-smoker	64 (70.3)	40 (71.4)	24 (68.6)	
Smoker	14 (15.4)	8 (14.3)	6 (17.1)	
Ex-smoker	13 (14.3)	8 (14.3)	5 (14.3)	
**Alcohol**, *n (%)*				0.117
No	65 (76.5)	36 (70.6)	29 (85.3)	
Yes	20 (23.5)	15 (29.4)	5 (14.7)	

Statistical significance was established as *p* < 0.05.

**Table 2:** Taste examination in patients with burning mouth syndrome (BMS).

	Response, Mean (SD)		*p*-Value
	No	Yes	
**Taste alteration**			
VAS_burning pain score (0–10)	7.85 (1.81)	8.07 (2.05)	0.743
Duration years	2.48 (2.56)	1.93 (1.71)	0.483
**Sweet**			
VAS_burning pain score (0–10)	9.67 (0.58)	7.78 (1.90)	0.1
Duration years	1.17 (0.76)	2.34 (2.29)	0.388
**Salty**			
VAS_burning pain score (0–10)	8.33 (2.08)	7.91 (1.91)	0.715
Duration years	1.50 (0.87)	2.31 (2.30)	0.552
**Acid**			
VAS_burning pain score (0–10)	7.57 (2.37)	7.72 (1.93)	0.871
Duration years	1.07 (0.53)	1.78 (0.97)	0.085
**Quinine Hydrochloride**			
VAS_burning pain score (0–10)	8.33 (2.06)	7.31 (1.96)	0.232
Duration years	1.39 (1.11)	1.69 (0.81)	0.448

VAS: visual analog scale; (0 = no pain, 10 = extreme pain) Statistical significance was established as *p* < 0.05.

## Data Availability

Not applicable.
